# Forecasting escalation of cardio-respiratory instability using noninvasive vital sign monitoring data

**DOI:** 10.1186/2197-425X-3-S1-A590

**Published:** 2015-10-01

**Authors:** M Guillame-Bert, A Dubrawski, L Chen, MT Hravnak, G Clermont, MR Pinsky

**Affiliations:** Carnegie Mellon University, School of Computer Science, Pittsburgh, PA USA; Carnegie Mellon University, Heinz College, Pittsburgh, PA USA; University of Pittsburgh, School of Nursing, Pittsburgh, PA USA; University of Pittsburgh, School of Medicine, Pittsburgh, PA USA

## Introduction

Critical cardio-respiratory instability (CRIcrit) poses a substantial risk for patients in step-down-units (SDU) and requires immediate medical attention. It is often preceded by mild episodes of CRI (CRImild).

## Objective

Apply Machine Learning (ML) to continuous noninvasive vital sign (VS) monitoring data of patients experiencing CRImild, and predict which of these patients will deteriorate to CRIcrit.

## Methods

Our data includes the continuously monitored VS (heart rate, respiratory rate, and SpO2, sampled every 20s, and blood pressure sampled every 2h on average) of 156 SDU patients whose VS exceeded criteria for CRImild (heart rate < 40 or >140, respiratory rate < 8 or >36, systolic BP < 80 or >200, diastolic BP>110, SpO2 < 85%) for either brief intervals or had questionable clinical significance [1]. Of these CRImild patients, 29 deteriorated to CRIcrit, which was more severe and generally displaying multiple VS abnormalities simultaneously at least 1h after the initial CRImild event. The remaining 127 patients had CRImild but never deteriorated to CRIcrit. For each patient, we extracted two partially overlapping 1h snapshots of VS data, one preceding the onset of CRImild event (A), and the other centered on it (B). Statistical features of the VS were extracted from each snapshot, and fed to a ML random forest classifier trained to predict if the patient will deteriorate to a CRIcrit 1h or later from the onset of the leading CRImild event. We quantified performance of the resulting models A and B using leave-one-patient-out cross validation.

## Results

**Figure 1:**
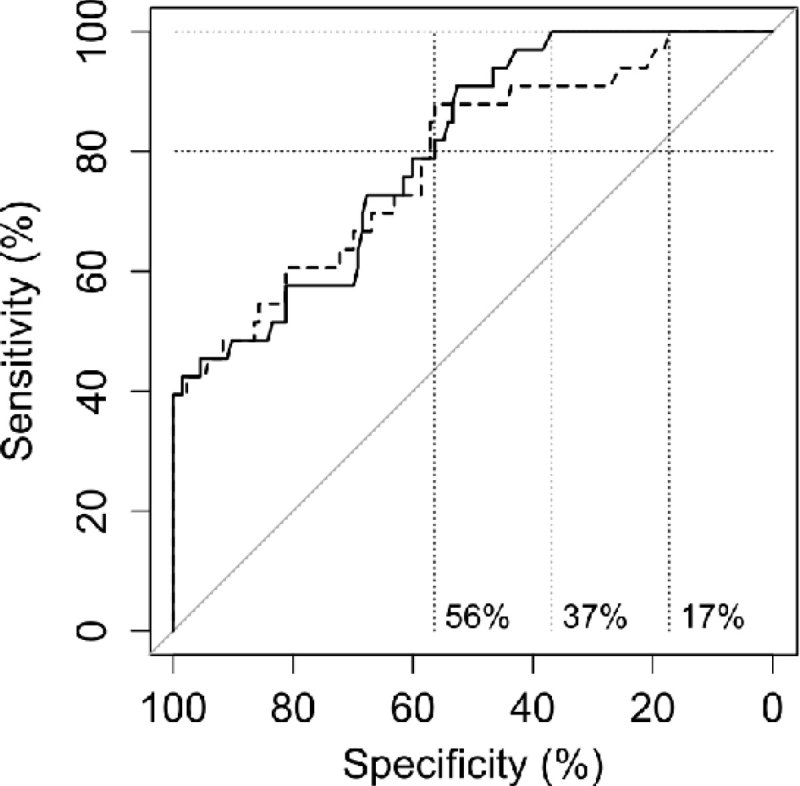
**ROC of Models A (dashed) and B (solid).**

Model A using data available at the onset of the leading CRImild event identifies 80% of patients who will deteriorate to CRIcrit with 56% specificity and 100% such patients at 17% specificity, achieving area under the ROC (AUC) score of 78% (Figure 1). Model B, which includes 30 min of data observed during and after the CRImild event boosts AUC to 80.1% and specificity at 100% sensitivity to 37% (identifies all patients at elevated risk of CRIcrit at least 30 minutes ahead of its onset, while correctly identifying 37% of those who will not experience such deterioration). Extending the observation period to 30 min after the initial CRImild more than doubles the ability to identify low risk of CRIcrit.

## Conclusions

ML can dynamically triage patients undergoing CRImild episodes to obtain a highly sensitive prediction of which of those patients will deteriorate to CRIcrit at least 30-60 min in advance, while confidently eliminating patients unlikely to deteriorate. These results suggest potential triage utility in assessing CRI.

## Grant Acknowledgements

NSF 1320347, NIH 1R01NR013912.
